# Theoretical Investigation of the Influence of Wavelength on the Bandwidth in Multimode W-Type Plastic Optical Fibers with Graded-Index Core Distribution

**DOI:** 10.3390/polym13223973

**Published:** 2021-11-17

**Authors:** Ana Simović, Svetislav Savović, Branko Drljača, Alexandar Djordjevich, Rui Min

**Affiliations:** 1 Faculty of Science, University of Kragujevac, R. Domanovića 12, 34000 Kragujevac, Serbia; asimovic@kg.ac.rs (A.S.); savovic@kg.ac.rs (S.S.); 2 Department of Mechanical Engineering, City University of Hong Kong, 83 Tat Chee Avenue, Hong Kong 7903, China; mealex@cityu.edu.hk; 3 Faculty of Sciences, University of Priština in Kosovska Mitrovica, Lole Ribara 29, 38220 Kosovska Mitrovica, Serbia; branko.drljaca@pr.ac.rs; 4 Center for Cognition and Neuroergonomics, State Key Laboratory of Cognitive Neuroscience and Learning, Beijing Normal University at Zhuhai, Zhuhai 519087, China

**Keywords:** Poly(methyl methacrylate) optical fiber, W-type plastic optical fiber, graded-index optical fiber, power flow equation, bandwidth

## Abstract

The bandwidth of multimode W-type plastic optical fibers (POFs) with graded-index (GI) core distribution is investigated by solving the time-dependent power flow equation. The multimode W-type GI POF is designed from a multimode single-clad (SC) GI POF fiber upon modification of the cladding layer of the latter. Results show how the bandwidth in W-type GI POFs can be enhanced by increasing the wavelength for different widths of the intermediate layer and refractive indices of the outer cladding. These fibers are characterized according to their apparent efficiency to reduce modal dispersion and increase bandwidth.

## 1. Introduction

In the last three decades, data traffic demand has increased exponentially for access and backbone networks. The growth was accelerated by streaming transmissions and cloud computing. This concept also becomes a strong approach for use in optical networking strategies for automotive communications systems, given the advances in both intelligent driving systems and multimedia services. These systems usually use multimode POFs (Poly(methyl methacrylate), or PMMA, optical fibers) thanks to their easy connection and strength in tight curves.

Transmission characteristics of multimode POFs strongly depend upon the differential mode-attenuation and mode coupling [1]. The latter represents power transfer between neighboring modes caused by fiber impurities and inhomogeneities introduced during the fiber manufacturing process (such as microscopic bends, irregularity of the core-cladding boundary and refractive index distribution fluctuations) [2]. Mode coupling increases fiber bandwidth in data networks by reducing modal dispersion [3]. In practice, when installing a POF-based link, the cable has to be repeatedly bent, thus increasing the strength of mode coupling and radiation losses [4]. It has been shown that organic glass-clad PMMA fibers show a similar strength of mode coupling compared to standard POFs but stronger mode coupling than do the plastic-clad silica fibers [5].

Studies have been reported using geometric optics (ray approximation) to investigate transmission in optical fibers [6,7]. By employing the time-independent power flow equation [2,8] as well as the Fokker–Planck and Langevin equations [9], far-field patterns in SI POFs have been predicted as a function of the launch conditions and fiber length. By employing the time-dependent power flow equation, the bandwidth of GI POF has been obtained for different radial launch conditions [10].

In terms of the number of functional cover layers, multimode optical fibers are usually SC fibers. Special detection [11], modulation [12], equalization [13] and compensation for modal-dispersion [14] can improve the bandwidth and attenuation properties of SC fiber. Since the structure of fiber can influence its features significantly, different POFs (double-clad fibers) of the step-index (SI), GI and W types were proposed [15,16,17]. Due to their progressively decreased core refractive index with a radial distance from the fiber axis, GI POFs have a much lower modal dispersion than SI POFs [18]. Since the dispersion of the W-type fiber is smaller than that of SC fiber [19], W-type fiber has a larger bandwidth and lower bending losses compared to a corresponding fiber of SC. The glass optical fiber’s bandwidth-distance product is approximately 30 MHz·km for SC and approximately 50 MHz·km for W-type fiber. The POFs have a bandwidth-distance product of approximately 15 MHz·km for the SC and approximately 200 MHz·km for the W-type fibers [15,17,20].

The intermediate layer of a W-type fiber (inner cladding) decreases the dispersion and enlarges the bandwidth, thereby lowering the number of guided modes that are held closer to the core [20,21]. Optical power is transferred between modes because of the mode coupling [2]. Methods are needed for calculating modal attenuation and coupling to loss modes of W-type fiber’s intermediate layer, and for optimizing the fiber’s refractive index profile in order to minimize the group delay difference between modes in the output field [20,22]. In this study, we examined how the wavelength influences the bandwidth for various W-type GI POF configurations. It should be noted that our calculations include modal attenuation, mode coupling and modal dispersion.

## 2. Time-Dependent Power Flow Equation

**Figure 1:**
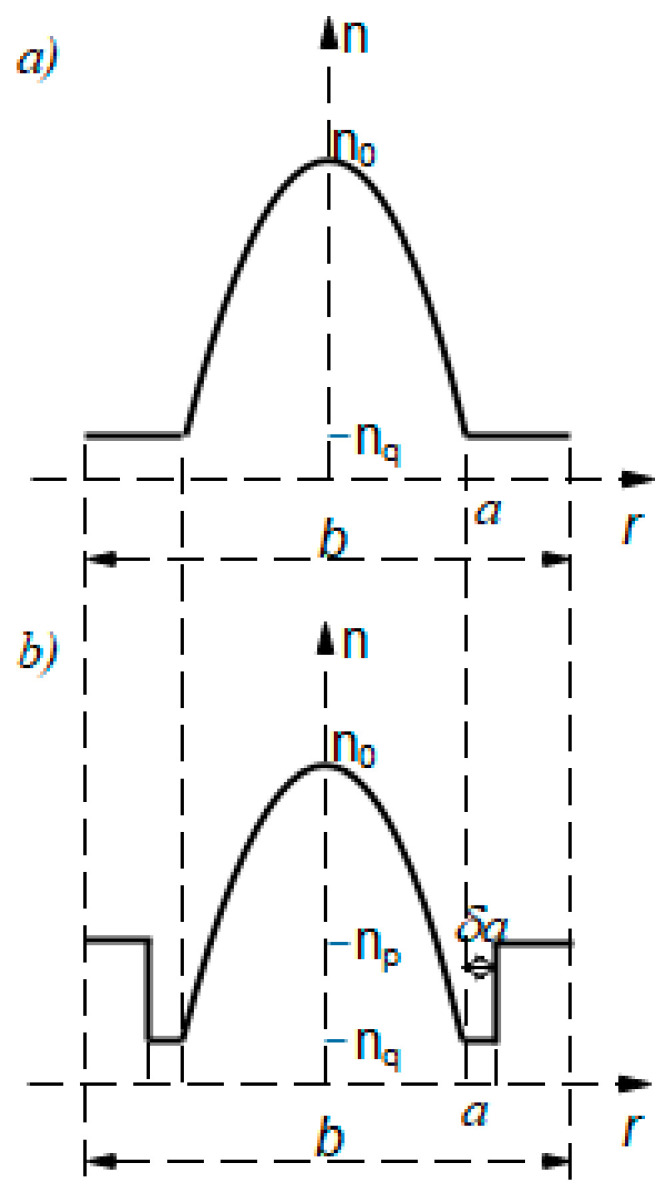
Refractive index profile of (**a**) SC GI POF and (**b**) W-type GI POF.

Consider a W-type GI fiber with refractive index profile, as shown in Figure 1b. The refractive index profile of W-type optical fibers with GI distribution of the core may be expressed as [8,23]:

(1) $$n(r,\lambda )=\left\{\begin{array}{l} n_{0}(\lambda ){\left[1-\Delta_{q}(\lambda ){\left(\frac{r}{a}\right)}^{g}\right]}^{1/2}\;\;\;\;(0\leq r\leq a)\\ n_{q}\;\;\;\;\;\;\;\;\;\;\;\;\;\;\;\;\;\;\;\;\;\;\;\;\;\;\;\;\;\;\;(a<r\leq a+\delta a)\\ n_{p}\;\;\;\;\;\;\;\;\;\;\;\;\;\;\;\;\;\;\;\;\;\;\;\;\;\;\;\;\;\;\;(a+\delta a<r\leq \frac{b}{2}) \end{array}\right\}$$

where g is the core index exponent, *a* is the core radius, *δa* is the intermediate layer width, *b* is the fiber diameter, *n*_0_(*λ*) is the maximum index of the core (measured at the fiber axis), *n_q_* and *n_p_* are refractive indices of the intermediate layer and cladding respectively, and $\Delta_{q}=(n_{0}-n_{q})/n_{0}$ is the relative index difference between the core and intermediate layer.

The time-dependent power flow equation for multimode optical fibers with GI core distribution is:

(2) $$\frac{\partial P(m,\lambda ,z,\omega )}{\partial z}+j\omega \tau (m,\lambda )P(m,\lambda ,z,\omega )=-\alpha (m,\lambda )P(m,\lambda ,z,\omega )+\;+\frac{\partial P(m,\lambda ,z,\omega )}{\partial m}\frac{\partial d(m,\lambda )}{\partial m}+d(m,\lambda )\frac{1}{m}\frac{\partial P(m,\lambda ,z,\omega )}{\partial m}+d(m,\lambda )\frac{\partial P^{2}(m,\lambda ,z,\omega )}{\partial m^{2}}$$

where *P*(*m,λ,z,ω*) is the power in the *m*—the principal mode (modal group), *z* is the coordinate along the fiber axis from the input fiber end, α(*m*,λ) = $\alpha_{0}+\alpha_{d}(m,\lambda )$ is the attenuation of the mode *m*, where *α*_0_ represents conventional losses due to absorption and scattering (the term *α*_0_ leads only to a multiplier *exp*(−*α_0_z*) in the solution and is thus neglected, the term $\alpha_{d}(m,\lambda )$ in the expansion of α(m) is dominant for higher-order modes), *d*(*m*,λ) is the coupling coefficient of the mode *m*, *ω = 2πf* is the baseband angular frequency and τ(m,λ) is delay time per unit length of mode *m*, which can be determined as:

(3) $$\tau (m,\lambda )≅\frac{n_{0}(\lambda )}{c}\left[1+\frac{g-2}{g+2}\Delta_{q}^{}(\lambda ){\left(\frac{m}{M(\lambda )}\right)}^{2g/(g+2)}+\frac{1}{2}\frac{3g-2}{g+2}\Delta_{q}{\left(\lambda \right)}^{2}{\left(\frac{m}{M(\lambda )}\right)}^{4g/(g+2)}\right]$$

where *c* is the free-space velocity of light and:

(4) $$P(m,\lambda ,z,\omega )=\int_{-\infty }^{+\infty }P(m,\lambda ,z,t)\exp(-j\omega t)dt$$

The maximum principal mode number, *M*(*λ*), can be obtained as [24]:

(5) $$M(\lambda )=\sqrt{\frac{g\Delta_{q}(\lambda )}{g+2}}akn_{0}(\lambda )$$

where  k=2π/λ is the free-space wave number. Gaussian launch-beam distribution, *P_0_*(*θ,*λ*,z =* 0), can be converted to *P_0_*(*m,*λ*,z =* 0) (one needs *P_0_*(*m,*λ*,z =* 0) to solve the power flow equation numerically (2)), using the following relationship [25]:

(6) $$\frac{m}{M(\lambda )}={\left[{\left(\frac{r_{0}}{a}\right)}^{g}+\frac{\theta_{}^{2}}{2\Delta_{q}(\lambda )}\right]}^{(g+2)/2g}$$

where *r*_0_ is radial distance (radial offset) between the launch beam position and the core center and *θ* is the propagation angle with respect to the core axis.

The relative refractive index difference, $\Delta_{q}=(n_{0}-n_{q})/n_{0}$, between the core and intermediate layer is larger than the difference $\Delta_{p}=(n_{0}-n_{p})/n_{0}$ between the core and cladding. Modes propagating along with subcritical angles *m* < *m_q_* are guided. The same is true for those propagating through the complete W-fiber, with *m* below the critical value *m_p_*. On the other hand, modes with angles between *m_p_* and *m_q_* ≡ *M* are transformed into leaky modes [10]. The leaky mode attenuation constants are provided as:

(7) $$\alpha_{L}(m,\lambda )=\frac{4\sqrt{2\Delta_{q}}{\left({\left(\frac{m}{m_{q}}\right)}^{\frac{2g}{g+2}}-{\left(\frac{m_{p}}{m_{q}}\right)}^{\frac{2g}{g+2}}\right)}^{1/2}}{a{\left(1-2\Delta_{q}{\left(\frac{m}{m_{q}}\right)}^{\frac{2g}{g+2}}\right)}^{1/2}}\frac{{\left(\frac{m}{m_{q}}\right)}^{\frac{2g}{g+2}}\left(1-{\left(\frac{m}{m_{q}}\right)}^{\frac{2g}{g+2}}\right)}{\left(1-\frac{\Delta_{p}}{\Delta_{q}}{\left(\frac{m_{p}}{m_{q}}\right)}^{\frac{2g}{g+2}}\right)}\exp\left[-2\delta an_{0}k{\left(2\Delta_{q}\left(1-{\left(\frac{m}{m_{q}}\right)}^{\frac{2g}{g+2}}\right)\right)}^{1/2}\right]$$

One can see from Equation (7) that for thick intermediate layer widths, δ, the lower leaky modes are substantially guided because of the low leaky modes’ losses. Experiments in a SC GI fiber reveal that attenuation remains constant throughout the guided-mode region (*m* ≤ $m_{p}$) and rises quite steeply in the radiation-mode region [26]. As a result, in a W-type GI POF, the modal attenuation can be expressed as:

(8) $$\alpha_{d}(m,\lambda )=\left\{\begin{array}{l} 0\;\;\;\;\;\;\;\;\;\;\;\;\;\;\;\;\;\;\;\;\;\;\;\;\;m\leq m_{p}\\ \alpha_{L}(m,\lambda )\;\;\;\;\;\;\;\;\;\;\;\;\;\;\;\;m_{p}<m<m_{q}\;\;\;\\ \infty \;\;\;\;\;\;\;\;\;\;\;\;\;\;\;\;\;\;\;\;\;\;\;\;m\geq m_{q} \end{array}\right.\;\;\;\;\;\;\;\;\;\;\;\;$$

A W-type fiber can be thought of as a combination of SCq fiber with cladding. In the SCq fiber, modes *m* < $m_{q}$ can be guided. When the SCq fiber is coupled with surrounding medium of index *n_p_*, the lower-order modes *m* < $m_{p}$ remain guided, while the higher-order modes *m* ($m_{p}<m<m_{q}$) are transformed into leaky modes. Due to the strong dependence of *α_L_*(*m*) on the intermediate layer width *δa* (Equation (7)), it is expected that characteristics of a W-type GI fiber also depend on *δa* and coincide with those of SCp and SCq GI fibers in the limits of *δ*→0 and *δ*→∞, respectively [20,27].

It is obvious that *P*(*m,*λ*,z,ω*) is complex. By separating *P* into the real part *P^r^* and imaginary part *P^i^*, Equation (2) can be rewritten as the following simultaneous partial differential equations:

(9) $$\frac{\partial P^{r}}{\partial z}=-\alpha P^{r}+\frac{\partial d}{\partial m}\frac{\partial P^{r}}{\partial m}+\frac{d}{m}\frac{\partial P^{r}}{\partial m}+d\frac{\partial^{2}P^{r}}{\partial m^{2}}+\omega \tau P^{i}$$

(10) $$\frac{\partial P^{i}}{\partial z}=-\alpha P^{i}+\frac{\partial d}{\partial m}\frac{\partial P^{i}}{\partial m}+\frac{d}{m}\frac{\partial P^{i}}{\partial m}+d\frac{\partial^{2}P^{i}}{\partial m^{2}}-\omega \tau P^{i}$$

where *P = P^r^ + jP^i^*. Assuming a constant coupling coefficient *d* ≡ *D*, Equations (9) and (10) can be written as:

(11) $$\frac{\partial P^{r}}{\partial z}=-\alpha P^{r}+\frac{D}{m}\frac{\partial P^{r}}{\partial m}+D\frac{\partial^{2}P^{r}}{\partial m^{2}}+\omega \tau P^{i}$$

(12) $$\frac{\partial P^{i}}{\partial z}=-\alpha P^{i}+\frac{D}{m}\frac{\partial P^{i}}{\partial m}+D\frac{\partial^{2}P^{i}}{\partial m^{2}}-\omega \tau P^{r}$$

Using the EFDM, discretization of Equations (11) and (12) leads to:

(13) $$P_{k,l+1}^{r}=(\frac{\Delta zD}{\Delta m^{2}}-\frac{\Delta zD}{2m_{k}\Delta m})P_{k-1,l}^{r}+(1-\frac{2\Delta zD}{\Delta m^{2}}-\alpha_{k}\Delta z)P_{k,l}^{r}+(\frac{\Delta zD}{2m_{k}\Delta m}-\frac{\Delta zD}{\Delta m^{2}})P_{k+1,l}^{r}+\frac{\omega n_{0}\Delta z}{2c}m_{k}^{2}P_{k,l}^{i}$$

(14) $$P_{k,l+1}^{i}=(\frac{\Delta zD}{\Delta m^{2}}-\frac{\Delta zD}{2m_{k}\Delta m})P_{k-1,l}^{i}+(1-\frac{2\Delta zD}{\Delta m^{2}}-\alpha_{k}\Delta z)P_{k,l}^{i}+(\frac{\Delta zD}{2m_{k}\Delta m}-\frac{\Delta zD}{\Delta m^{2}})P_{k+1,l}^{i}-\frac{\omega n_{0}\Delta z}{2c}m_{k}^{2}P_{k,l}^{r}$$

where k and l refer to the discretization step lengths Δ*m* and Δ*z* for the mode *m* and length *z* respectively, i.e., $P_{k,l}^{r}\equiv P^{r}(m_{k},z_{l},\omega )$ and $P_{k,l}^{i}\equiv P^{i}(m_{k},z_{l},\omega )$.

If *P^r^* and *P^i^* are obtained by solving Equations (13) and (14), the transmission characteristics can be calculated. Thus, the frequency response of fiber at length *z* is:

(15) $$H(\lambda ,z,\omega )=\frac{\int_{1}^{M}2m\left[P_{r}(m,\lambda ,z,\omega )+jP_{i}(m,\lambda ,z,\omega )\right]dm}{\int_{1}^{M}2m\left[P_{r}(m,\lambda ,z,0)+jP_{i}(m,\lambda ,z,0)\right]dm}$$

where the factor 2*m* denotes degeneracy of modal group *m*. The frequency responses for a specific fiber can be determined over a wide range of lengths, revealing the bandwidth dependence with distance. The modal power distribution, $P_{F}^{}(m,\lambda ,z,\omega )$, and the spatial transient of power, $P_{L}^{}(\lambda ,z,\omega )$, can be obtained by:

(16) $$P_{F}^{}(m,\lambda ,z,\omega )={\left[P_{}^{r}{\left(m,\lambda ,z,\omega \right)}^{2}+P_{}^{i}{\left(m,\lambda ,z,\omega \right)}^{2}\right]}^{1/2}$$

(17) $$P_{L}(\lambda ,z,\omega )=2\pi \int_{0}^{M}mP_{F}(m,\lambda ,z,\omega )dm$$

One should note here that the numerical solution of the time-dependent power flow equation as well as the calculation of the frequency response and bandwidth in the analyzed W-type GI POF have been obtained by employing our FORTRAN90 code. This enabled us to determine the relationship between the transmission length and bandwidth in the W-type GI POF.

## 3. Numerical Results and Discussion

In this research, we investigated the bandwidth at various wavelengths in a differently constructed W-type GI POF designed from the SC GI POF (Figure 1a), which we experimentally investigated in our previously published works [8,10]. For the structure shown in Figure 1b, a W-shape refractive index model is adopted. SC GI POF (OM Giga, Fiber FinTM) had the following characteristics: The fiber’s core diameter was 2*a* = 0.9 mm (fiber diameter was *b* = 1 mm), the core’s refractive index measured along the fiber axis was *n*_0_ = 1.522 and the intermediate layer (inner cladding) cladding’s refractive index was *n*_q_ = 1.492. The maximum principal mode number for the examined SC GI POF was *M* = 656 at wavelength *λ* = 633 nm, *g* = 1.80101 and ∆*_q_*= (*n*_0_ − *n_q_*)/*n*_0_ = 0.019711. The W-type GI POF with GI distribution of the core is designed from this SC GI POF in such a way that the W-type GI POF’s inner cladding retains the distribution of the SC GI POF’s cladding, while the W-type GI POF’s outer cladding has a refractive index, *n_p_*, that is higher than the inner cladding’s refractive index, *n_q_* (Figure 1b). In the modeling, three values of the outer cladding refractive index, *n_p_*, were used: *n_p_* = 1.51366 (*m_p_* = 346), *n_p_* = 1.51065 (*m_p_* = 404) and *n_p_* = 1.50718 (*m_p_* = 461). The normalized intermediate layer widths of *δ* = 0.001, *δ* = 0.002 and *δ* = 0.003 (actual width is *δ·a* mm) were used. In the calculations, the constant coupling coefficient d(m,λ) ≡ D=1482   1/m was employed [8]. At *λ* = 476, 522, 568 and 633 nm, the maximum principal mode number in such proposed W-type GI POFs is *M* = 872, 795, 731 and 656, respectively. In the numerical calculations, a Gaussian beam, *P*(*θ,z*), is assumed to be launched with 〈*θ*〉 = 0° and standard deviation *σ_θ_* = 1.3° (FWHM = 3.06°). The width of the launched beam measured at the end of a single-mode optical fiber, which is butt-coupled to the SC GI POF examined in our earlier experimental work [8], is described by this standard deviation. The numerical calculations have been performed for radial offset ∆*r* = 0 µm.

**Figure 2:**
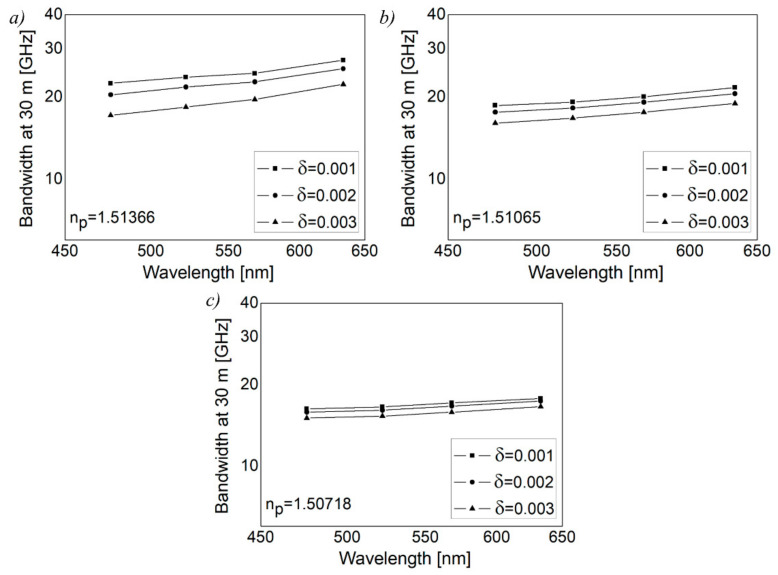
Numerical results for bandwidth for W-type GI POF at 30 m as a function of wavelength for (**a**) *n_p_* = 1.51366, (**b**) *n_p_* = 1.51065 and (**c**) *n_p_* = 1.50718, where (FWHM)_z=0_ = 3.06°, δ = 0.001, 0.002 and 0.003 and D=1482   1/m.

**Figure 3:**
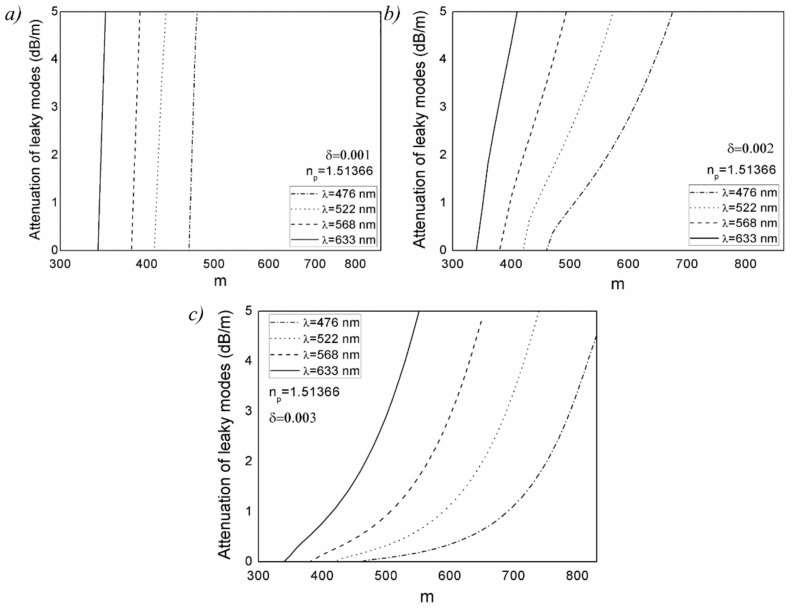
Leaky mode losses for W-type GI POF as a function of wavelength, for (**a**) *δ* = 0.001, (**b**) *δ* = 0.002 and (**c**) *δ* = 0.003, and *n_p_* = 1.51366.

Figure 2 shows our numerical solution to the time-dependent power flow equation. It depicts the evolution of the W-type GI POF’s bandwidth at 30 m with wavelength for varied widths of intermediate layer, *δ*, in three inserts corresponding to three outer cladding refractive indices: *n_p_* = 1.51366 (*m_p_* = 346), *n_p_* = 1.51065 (*m_p_* = 404) and *n_p_* = 1.50718 (*m_p_* = 461). Figure 2 shows that the influence of wavelength on bandwidth is less noticeable for the smallest width of the intermediate layer (*δ* = 0.001). This is due to large leaky mode losses (Figure 3), and as these modes are practically not guided along the fiber, modal dispersion (bandwidth) changes very little. There is a wavelength-dependent drop in bandwidth as the width of the intermediate layer increases. At short wavelengths, this decrease is more pronounced because leaky mode losses are reduced (Figure 3), resulting in a rise in modal dispersion and a fall in fiber bandwidth.

When the refractive index of the outer cladding, *n_p_*, is increased, leaky mode losses increase, resulting in the maximum bandwidth values in the case of *δ* = 0.001. The improvement in the bandwidth of W-type GI POFs is more noticeable at longer fiber lengths, as seen in Figure 2. Since a narrow launch beam distribution is assumed in the calculations, only guided modes are excited at the input fiber length. As a result, for short fiber lengths, leaky modes play a less important role. More leaky modes are filtered out with longer fiber lengths due to mode coupling, resulting in the significant improvement of the bandwidth of the W-type GI POF [28].

## 4. Conclusions

For W-type GI POFs with a variable width of the intermediate layer and refractive index of the outer cladding, bandwidth was determined using the time-dependent power flow equation over a variety of light wavelengths. The bandwidth of W-fibers broadened with larger wavelengths for all intermediate layer widths, as shown in this study. Higher leaky mode losses and the resulting decline in modal dispersion caused this broadening of the bandwidth. Since leaky mode losses grow as the outer cladding’s refractive index rises, the bandwidth rises as well. These findings can be used to design and implement a W-type GI POF at various operating wavelengths.

## Data Availability

The data presented in this study are available on request from the corresponding author.
